# Pharmacokinetics and bioequivalence study of esomeprazole magnesium enteric-coated tablets 20 mg in healthy Chinese subjects under fasting and fed conditions

**DOI:** 10.3389/fphar.2023.1169103

**Published:** 2023-04-28

**Authors:** Ying Ding, Nannan Chu, Linling Que, Kai Huang, Yuanxing Chen, Wei Qin, Zhenzhong Qian, Yunfei Shi, Zhen Xu, Qing He

**Affiliations:** Drug Clinical Trial Institution, Wuxi People’s Hospital Affiliated with Nanjing Medical University, Wuxi, Jiangsu, China

**Keywords:** esomeprazole magnesium enteric-coated tablet, bioequivalence, pharmacokinetics, safety, healthy Chinese subjects

## Abstract

**Objective:** The main purpose of this study was to evaluate the pharmacokinetics, bioequivalence, and safety properties between a new generic and a brand reference formulation of esomeprazole enteric-coated tablets 20 mg in healthy Chinese subjects under fasting and fed conditions.

**Methods:** The fasting study was an open-label, randomized, two-period crossover study conducted in 32 healthy Chinese volunteers, and the fed study was a four-period crossover study conducted in 40 healthy Chinese volunteers. Blood samples were collected at the specified time points and determined to obtain the plasma concentrations of esomeprazole. The primary pharmacokinetic parameters were calculated using the non-compartment method. Bioequivalence was analyzed by the geometric mean ratios (GMRs) of the two formulations and the corresponding 90% confidence intervals (CIs). The safety of the two formulations was assessed.

**Results:** The fasting and fed study showed that the pharmacokinetics of the two formulations was similar. Under the fasting condition, the 90% CIs of GMRs of the test-to-reference formulation were 87.92%–104.36% for C_max_, 87.82%–101.45% for AUC_0-t_, and 87.99%–101.54% for AUC_0-∞_; under the fed condition, the 90% CIs of GMRs of the test-to-reference formulation were 80.53%–94.95% for C_max_, 87.46%–97.26% for AUC_0-t_, and 87.46%–97.16% for AUC_0-∞_. The 90% CIs of GMRs fall within the bioequivalence range of 80.00%–125.00%. The two formulations had good safety and were well-tolerated, and no serious adverse events occurred.

**Conclusion:** According to relevant regulatory standards, esomeprazole enteric-coated generic and reference products exhibited bioequivalence and good safety in healthy Chinese subjects.

**Clinical Trials Registration:** http://www.chinadrugtrials.org.cn/index.html, identifier CTR20171347 and CTR20171484

## Introduction

Gastroesophageal reflux disease (GERD) refers to the discomfort and/or complications caused by the flow of stomach contents back into the esophagus; heartburn and reflux are the typical symptoms (Vakil N et al., 2006). GERD has high morbidity, which is increasing gradually. The incidence rate of GERD in economically developed countries is about 15%–25%, which is higher than that in most economically underdeveloped countries (<10%) (El-Serag et al., 2014; Richter et al., 2018). GERD can lead to a decline in quality of life, and its recurrence and the requirement of long-term treatment can require a large number of medical resources and bring high costs to society (Kulig et al., 2003; Becher et al., 2011).

Effective control of the symptoms of GERD, prevention of the complications of GERD, and improvement of the patient’s quality of life are the main goals of GERD treatment (Dickman et al., 2015). At present, in addition to histamine type 2 receptor antagonists (H2RAs) commonly used in the clinic, proton pump inhibitors (PPIs) are the first-line drugs for GERD treatment. Research and development of PPIs have completely revolutionized the management of acid reflux. PPIs can significantly inhibit gastric acid secretion, improve the healing rate of the esophageal mucosa, and effectively control GERD symptoms (Gralnek et al., 2006).

Esomeprazole is the S-isomer of omeprazole and the first single optical isomer in the PPI family. Compared with other PPI drugs, esomeprazole has higher systemic bioavailability because it has hepatic first-pass metabolic effect and lower plasma clearance rate (Lindberg P et al., 2003). Many clinical studies have proved that esomeprazole can further improve the efficacy for GERD and other diseases related to gastric acid secretion, including gastric and duodenal ulcer, non-erosive reflux disease (NERD), and Zollinger–Ellison syndrome (Mckeage et al., 2008).

Because esomeprazole is unstable in an acidic environment, it is taken orally in the form of enteric-coated tablets. Currently, esomeprazole enteric-coated tablets at 20 mg and 40 mg, developed by AstraZeneca UK Limited, obtained marketing approval in over 125 countries. Some articles have reported the pharmacokinetic profile of esomeprazole (Hassan-Alin et al., 2000; Andersson et al., 2001). After administering 20 mg or 40 mg of enteric-coated tablets, the plasma concentrations of esomeprazole reach the peak value (C_max_) within 1–2 h (Hassan-Alin et al., 2005). The absolute bioavailability of 40 mg esomeprazole after single oral or repeated daily administration was 64% and 89%, respectively (Hassan-Alin et al., 2005). The absolute bioavailability of 20 mg of a single dose and multiple doses was 50% and 68%, respectively, which was less than that of 40 mg. The human plasma protein binding rate of esomeprazole was 97% *in vitro*. Although food intake can delay absorption and reduce the exposure of esomeprazole in the human body, it has no substantial effect on inhibiting the gastric acid secretion of esomeprazole (AstraZeneca UK Limited, 2022; AstraZeneca UK Limited, 2022). Esomeprazole is mainly metabolized by cytochrome P450 (CYP) 2C19 and CYP3A4 in the liver, most of which are metabolized by CYP2C19 to hydroxy and desmethyl metabolites, and a small part is converted from CYP3A4 to sulfone metabolites (Abelo et al., 2000). The average plasma elimination half-life (t_1/2_) of a single dose of esomeprazole 20 or 40 mg in healthy subjects was 0.75 and 0.85 h, respectively. On the fifth day, after repeated administration of esomeprazole 20 or 40 mg, the t_1/2_ of esomeprazole was extended to 1.01 h and 1.25 h, respectively (Hassan-Alin et al., 2000). After taking esomeprazole orally, most of the drug is excreted in the form of inactive metabolites, of which about 80% is excreted through urine, and a small part is excreted through feces (Andersson et al., 2001). Only a very small amount (<1%) of the parent drug is found in urine (Andersson et al., 2001). The pharmacokinetic difference of esomeprazole in populations with poor metabolism (i.e., the population lacking a functional CYP2C19 enzyme) had no significant clinical significance (Andersson et al., 2001). In addition, the pharmacokinetics of esomeprazole had no clinically significance in women versus men (Andersson et al., 2001), in the elderly (Hasselgren et al., 2001), and in patients with renal impairment (Naesdal et al., 1986) or mild-to-moderate hepatic impairment (Sjovall et al., 2002), so these population do not need to adjust the dosage (AstraZeneca UK Limited, 2022).

The two dosages (40 mg and 20 mg) of esomeprazole enteric-coated tablet (Nexium^®^, AstraZeneca UK Limited) were approved and listed in China in 2003. Until now, only one paper reported the pharmacokinetics of esomeprazole enteric-coated tablets in Chinese subjects, and the administration dosage in the study was 40 mg (Liu et al., 2020), which indicated that the pharmacokinetic data of esomeprazole enteric-coated tablets at 20 mg were lacking in the Chinese population. In addition, due to the difficulty of pharmaceutical preparations, there were no domestic generic drugs of 20 mg esomeprazole enteric-coated tablets marketed in China before this study. Recently, a generic esomeprazole enteric-coated tablet at 20 mg was researched by Jiangxi Shanxiang Pharmaceutical Co., Ltd. (Jiangxi, China). In order to meet the requirements of generic drug listing in China, we compared the bioavailability of the generic esomeprazole enteric-coated tablet at 20 mg with that of the original product (Nexium^®^, AstraZeneca UK Limited). According to the guidelines related to bioequivalence, two studies under fasting and feeding conditions need to be carried out in healthy Chinese subjects. The bioequivalence results obtained in the study will provide the listing support for the generic drug in China.

## Methods

### Study drugs

The 20 mg esomeprazole enteric-coated tablet used as the test product was produced and provided by Jiangxi Shanxiang Pharmaceutical Co., Ltd. (Batch No. 170701, valid until June 2019). Meanwhile, a 20 mg esomeprazole enteric-coated tablet manufactured by AstraZeneca UK Limited (Nexium^®^, Batch No. MW325, valid until June 2019) was chosen as the reference product, which was also provided by Jiangxi Shanxiang Pharmaceutical Co., Ltd. In each treatment period, each subject received the test preparation or reference preparation of esomeprazole enteric-coated tablet 20 mg.

### Subjects

Eligible subjects needed to meet the following basic criteria: both men and women, aged over 18 years, weight of male subjects greater than 50 kg, weight of female subjects greater than 45 kg, and body mass index (BMI) within the range of 19.0–26.0 kg/m^2^. Clinical investigators introduced the study’s purpose, procedure, and potential risks to each subject in detail, and each one voluntarily signed an informed consent form after full consideration before participating in the study. After signing the informed consent forms, all enrolled subjects underwent a series of inquiries and examinations, including examination of demographic data, past medical history, physical examination, vital signs, clinical laboratory tests, serum immunovirological examination, a 12-lead electrocardiogram (ECG), X-ray of chest, and abdomen B-ultrasound evaluation. If these examination results were normal or abnormal but clinically insignificant, judged by research doctors, subjects were considered healthy and allowed to participate in the study.

Subjects with a history of severe digestive tract diseases or allergies to esomeprazole or its analogs were excluded because these abnormalities may potentially affect the absorption or disposition of esomeprazole. If subjects donated blood or had acute blood loss (blood volume of more than 450 mL) in the past 3 months, they were excluded. Subjects were excluded if they had a history of taking medicine within 30 days prior to screening, this covered all medicines including Chinese herbal medicine and or vitamin products, or if they took part in other clinical trials within 3 months prior to screening.

Meanwhile, lactating women or female subjects testing positive for pregnancy before enrollment were not allowed to participate in the study. All subjects had no fertility planning, and they and their partners were willing to take effective contraception and were not involved in egg or sperm donation programs.

### Study design

This study was conducted in the Phase I Clinical Research Center of Wuxi People’s Hospital Affiliated with Nanjing Medical University (Chinese Clinical Trial Registry, Registration Nos CTR20171347 and CTR20171484; http://www.chinadrugtrials.org.cn/index.html) following the guidelines of Good Clinical Practice (GCP) and the ethical principles of the Declaration of Helsinki. The relative study documents, including study protocol, informed consent form, and other files, were reviewed and approved by the independent ethics committee of Wuxi People’s Hospital Affiliated with Nanjing Medical University with the approval number 2017LLPJ-I-17 (Wuxi, Jiangsu, China).

The study consisted of two mutually independent clinical trials, including a fasting bioequivalence study and a feeding bioequivalence study. Regarding study design, according to the within-subject variability of esomeprazole, we comprehensively evaluated how many periods of cross-over tests should be carried out. Some reported references (Cai et al., 2011; Ullah et al., 2010; Sostek et al., 2007) showed that the within-subject coefficient of variation for (CV_w_%) esomeprazole under fasting and fed conditions were about 20% and 40%, respectively. Hence, we adopted the study design of a two-period crossover in the fasting study and a four-period crossover in the fed study. In terms of sample size estimation, we set CV_w_% of C_max_ and AUC_0-∞_ for esomeprazole to 20% and 40%, respectively; the significance level to 0.05; the power of the test to 0.8; the GMR of the two formulations to 0.95; and the equivalence threshold to 80.00%–125.00%. After calculation, we estimated the sample size to be 26 and 34 for the two trials, respectively. In addition, considering the possibility of early withdrawal of individual subjects and a drop-off rate of 20%, we finally enrolled 32 and 40 subjects to participate in the two trials.

### Fasting bioequivalence study

The fasting study adopted an open-label, randomized, two-formulation, two-period crossover, single-dose study design. In total, 32 enrolled subjects were admitted to the Phase I ward the day before administration. After completing the relevant examination before check-in, according to the random number table generated by SAS software (version 9.4), 32 subjects were randomly divided into the TR or RT groups in a ratio of 1:1. After fasting for at least 10 h overnight, subjects in the TR group received the test preparation and the reference preparation, respectively, in two treatment periods, while subjects in RT group took the opposite. The washout period between the two treatment periods was 7 days. After each administration, the subjects underwent a thorough oral examination to confirm they had taken the drug. The subjects were forbidden to drink water within 1 h prior to and after administration and needed to fast for at least 4 h after administration before they accepted standard lunch. The safety assessment was conducted throughout the study.

### Fed bioequivalence study

The fed study adopted an open-label, randomized, two-product, four-period crossover, single-dose study design. On the day before administration, 40 enrolled subjects were admitted to the Phase I ward. After completing the relevant examination before check-in, according to the random number table also generated by SAS software (version 9.4), 40 subjects were randomly divided into the TRTR group or RTRT group in a ratio of 1:1. The washout period between each treatment period was also 7 days. After fasting for at least 10 h overnight, the subjects started the standard high-fat and high-caloric breakfast 30 min before administration and completed it within 30 min. The calorific value of a standard high-fat breakfast was about 800–1000 kcal, including 150 kcal of protein, 250 kcal of carbohydrates, and 500–600 kcal of fat. Then, the subjects followed the same study procedure as in the fasting study.

### Pharmacokinetic sample collection and analysis methods

Vacuum tubes (4 mL) with EDTA-K_2_ anticoagulant were used to collect blood samples for detecting plasma concentrations. The time points for blood sample collection for the two trials were slightly different. In the fasting study, the time points for blood sample collection were 0 h (predose) and 20 min, 40 min, 1, 1.25, 1.5, 1.75, 2, 2.25, 2.5, 3, 3.5, 4, 5, 6, 8, 10, 12, and 24 h after administration. In the fed study, the time points for blood sample collection were 0 h (predose) and 30 min, 1, 1.5, 2, 2.5, 3, 3.5, 4, 4.5, 5, 5.5, 6, 7, 8, 10, 12, and 24 h after administration. After blood collection, sample centrifugation needed to be completed within 1 h, and the centrifugation condition was 2,152 g 4°C for 10 min. Then, the plasma was added separately into two tubes containing 30-µL carbonate buffer ( pH 8.50 ±0.05). The volume of plasma in the detection tube should be 1 mL, and the remaining plasma was transferred into a backup tube. The detection tube samples were kept frozen at −80°C in the clinical study center until these samples were transported to the analysis laboratory (Shanghai PharmaTech Co. Ltd., Shanghai, China). The backup tube samples were stored at −80°C in the clinical study center so as to provide available plasma samples when re-analysis was required.

The plasma samples were determined using HPLC-MS/MS methods. Before determining the plasma samples, the analytical method had been fully verified by linearity, sensitivity, specificity, intra- and inter-batch precision, accuracy, recovery, matrix effect, and stability. The standard curve range of esomeprazole was set to 3.0–3,000 ng/mL.

### Pharmacokinetic and statistical analysis

We used the non-compartmental method to analyze pharmacokinetics, and the analysis software used was Phoenix WinNonlin software (version 8.2) from Pharsight Corporation (California). The primary pharmacokinetic parameters included C_max_, the time of reach to C_max_ (T_max_), the area under the plasma concentration–time curve from time 0 to the time of the last measurable concentration (AUC_0-t_), the area under the plasma concentration–time curve from time 0 to infinity (AUC_0-∞_), and the terminal elimination half-life (t_1/2_). C_max_ and T_max_ were the actual observed measure values without analysis and calculation. AUC_0-t_ was calculated using the linear/log trapezoidal method. AUC_0-∞_ was calculated as AUC_0-∞_ = AUC_0-t_ + Ct/λz, where Ct was the last detectable concentration and λz was the elimination rate constant. λz was estimated using linear least-squares regression analysis for the concentration–time data obtained from the terminal log-linear phase. The results of t_1/2_ were obtained by dividing 0.693 by λz.

For the fasting study, an analysis of variance (ANOVA) was performed to compare the log-transformed pharmacokinetic parameters (C_max_, AUC_0-t_, and AUC_0-∞_) between the two formulations. The bioequivalence was evaluated by calculating the 90% CIs of the geometric least-squares mean (GLSM) ratio of the test preparation to reference preparation. If these 90% CIs were within the range of 80.00%–125.00%, the two formulations were considered bioequivalent.

For the fed study, the log-transformed pharmacokinetic parameters (C_max_, AUC_0-t_, and AUC_0-∞_) were also analyzed using ANOVA between the two formulations. Unlike the fasting study, in the fed study, we calculated the within-subject standard deviation (Swr) of C_max_, AUC_0-t_, and AUC_0-∞_. Swr was determined by the pharmacokinetic parameters of the reference formulation, and the value of Swr was determined for which the bioequivalence evaluation method was selected. For C_max_, AUC_0-t_, and AUC_0-∞_, (1) if Swr <0.294, average bioequivalence (ABE) was selected for analysis. Two one-sided *t*-tests with *α* = 0.05 were used to test the statistical hypothesis. Whether the 90% CIs of GLSM ratios of C_max_, AUC_0-t_, and AUC_0-∞_ of the test and reference formulation were within the range of 80.00%–125.00% determined if the two formulations were bioequivalent; and (2) if Swr ≥0.294, the reference-scale average bioequivalence (RSABE) was selected for analysis. The two formulations were considered bioequivalent if the statistical results met the following two criteria at the same time: (a) the upper bound of 95% CI of the test and reference formula (criteria bound) was less than or equal to 0, and (b) the point estimates of GMR of C_max_, AUC_0-t_, and AUC_0-∞_ of the test to reference formulation fall within the range of 80.00%–125.00%.

$$Formula:{\left({\bar{Y}}_{T},{\bar{Y}}_{R}\right)}^{2}-\theta S_{wr}^{2}.$$ (1)

### Safety assessment

During the study, safety was evaluated through clinical observation monitored by research doctors and adverse events (AEs) reported spontaneously by volunteers. At predefined time points, all subjects received vital signs, physical examinations, 12-lead ECG, and clinical laboratory tests. Vital signs were measured within 1 h before administration and 2, 8, and 24 h after administration in each treatment period. Clinical laboratory tests, 12-lead ECG, and physical examinations were conducted at the screening and end of the trial. For standardization of the AE report, we coded the name of all AEs in terms of the Medical Dictionary of Regulatory Activities (MedDRA^®^)and graded the severity of these AEs in accordance with the Common Terminology Criteria for Adverse Events (CTCAE, Version 5.0) published by the National Cancer Institute of the United States.

## Results

### Study population

**FIGURE 1:**
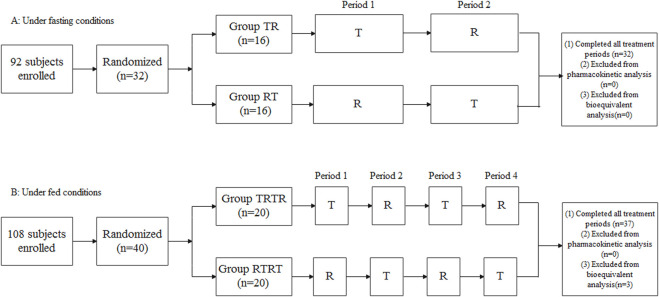
Study design and disposition of subjects. **(A)** Under fasting conditions, **(B)** Under fed conditions.

Figure 1 shows the study design and disposition of subjects in the two trials. In total, 92 and 108 healthy Chinese adult subjects in the two studies signed informed consent forms and participated in screening. In total, 32 and 40 healthy adult subjects met the enrollment criteria and were enrolled in the two studies. The fasting study included 21 male (65.6%) and 11 female subjects (34.4%), age 30.6 ± 8.97, and BMI 22.7 ± 2.11 kg/m^2^, and the fed study included 31 male (77.5%) and nine female subjects (22.5%), age 29.0 ± 7.15, and BMI 22.8 ± 1.63 kg/m^2^. The baseline demographic characteristics of the study population are shown in Table 1 **.** A total of 32 subjects completed the whole study under fasting conditions, and 37 subjects completed four treatment periods under fed conditions, except for three subjects who withdrew from the study due to AEs in different treatment periods.

**TABLE 1:** Baseline demographics and characteristics of study population.

Variable	Fasting study (*n* = 32)	Fed study (*n* = 40)
Sex		
Male (%)	21 (65.6%)	31 (77.5%)
Female (%)	11 (34.4%)	9 (22.5%)
Age, years		
Mean (SD)	30.6 (8.97)	29.0 (7.15)
Min–max	19–54	20–54
Height, cm		
Mean (SD)	165.2 (7.20)	169.1 (9.28)
Min–max	152.9–180.7	149.7–190.9
Weight, kg		
Mean (SD)	62.2 (9.27)	65.4 (8.83)
Min–max	47.2–82.0	46.6–82.0
BMI, kg/m^2^		
Mean (SD)	22.7 (2.11)	22.8 (1.63)
Min–max	19.5–25.8	20.1–25.9

Notes: BMI, body mass index; SD, standard deviation; max, maximum; min, minimum.

### Pharmacokinetics and bioequivalence

**FIGURE 2:**
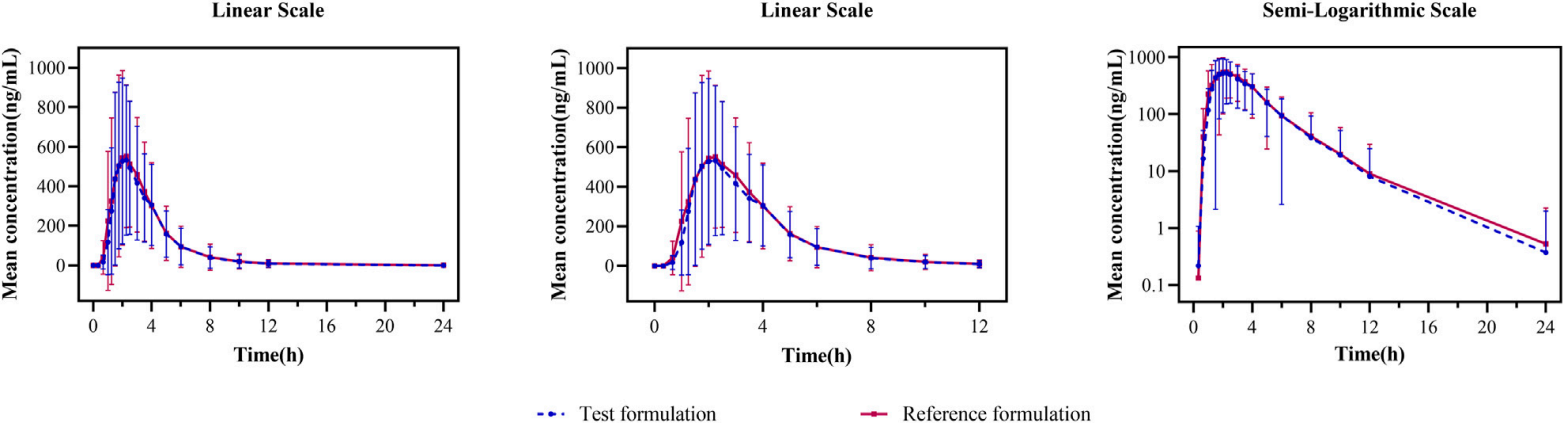
Mean plasma concentration–time curves for the test and reference formulations after a single dose of 20 mg of the esomeprazole enteric-coated tablet in healthy subjects under fasting conditions. *N* = 32. Bars represent SD. T: test formulation; R: reference formulation.

In the fasting study, the data of 32 enrolled subjects were entered into the pharmacokinetic analysis. Figure 2 shows the mean plasma concentration–time curves of the two formulations under fasting conditions. The changing trend of the two plasma concentration–time curves is very similar. The primary pharmacokinetic parameters are listed in Table 2. For the test or reference formulation, C_max_ were 769.13 ± 322.72 and 808.03 ± 354.40 ng/mL, AUC_0-t_ were 1809.10 ± 1248.65 and 1934.00 ± 1412.94 ng h/m, AUC_0-∞_ were 1827.56 ± 1270.32 and 1946.32 ± 1421.02 ng h/m, and t_1/2_ were 1.27 ± 0.64 and 1.25 ± 0.64 h. The results of bioequivalence assessment of the fasting study are shown in Table 3. C_max_, AUC_0-t_, and AUC_0-∞_ had no period, sequence, or formulation effect. The 90% CIs of the GLSM ratios of the test to the reference formulation were 87.92%–104.36% for C_max_, 87.82%–101.45% for AUC_0-t_, and 87.99%–101.54% for AUC_0-∞_. The 90% CIs were within the accepted bioequivalence range of 80.00%–125.00%, which suggested that the two formulations of esomeprazole exhibited bioequivalence under fasting conditions. The CV_w_% for C_max_, AUC_0-t_, and AUC_0-∞_ were 20.41%, 17.13%, and 17.00%, respectively, indicating that esomeprazole has no high variability from the perspective of pharmacokinetics. The powers of C_max_, AUC_0-t_, and AUC_0-∞_ were >95%, proving that our expected sample size is enough and reasonable.

**TABLE 2:** Pharmacokinetic parameters of esomeprazole after a single dose 20 mg of test and reference esomeprazole magnesium enteric-coated tablet in healthy Chinese subjects under fasting and fed conditions.

Pharmacokinetic parameters	Fasting condition (*N* = 32)		Fed condition (*N* = 40)	
	Test (*n* = 32)	References (*n* = 32)	Test (*n* = 78)	References (*n* = 76)
T_max_ (h)	2.00 (1.25–5.00)	2.00 (1.00–4.00)	3.50 (1.00–7.00)	3.50 (1.50–6.00)
C_max_ (ng/mL)	769.13 ± 322.72	808.03 ± 354.40	425.07 ± 220.29	467.04 ± 228.53
AUC_0-t_ (ng·h/mL)	1809.10 ± 1248.65	1934.00 ± 1412.94	1153.56 ± 746.72	1222.44 ± 758.19
AUC_0-∞_ (ng·h/mL)	1827.56 ± 1270.31	1946.32 ± 1421.02	1167.32 ± 760.77	1238.59 ± 775.71
t_1/2_ (h)	1.27 ± 0.64	1.25 ± 0.64	1.04 ± 0.40	1.01 ± 0.36

Notes: AUC_0-t_, area under the plasma concentration–time curve from time 0 to the time of the last measurable concentration; AUC_0-∞_, area under the plasma concentration–time curve from time 0 to infinity; C_max_, maximum plasma drug concentration; T_max_, time to reach C_max_; t_1/2_, half-time of terminal elimination. All values are expressed as mean ± SD except for T_max_ values, which are expressed as median (range). N, the pharmacokinetic analysis set population; n, the statistical analysis population.

**TABLE 3:** Bioequivalence assessment of pharmacokinetic parameters of esomeprazole after a single dose 20 mg of test and reference esomeprazole magnesium enteric-coated tablet in healthy Chinese subjects under fasting conditions.

Parameters	Fasting condition (*N* = 32)					
	GLSM T	GLSM R	Ratio of GLSM (%)	90% CI (%)	Intra-subject CV (%)	Power (%)
C_max_	700.765	731.600	95.79	87.92–104.36	20.41	96.98
AUC_0-t_	1476.2933	1564.0324	94.39	87.82–101.45	17.13	98.62
AUC_0-∞_	1488.6476	1574.8658	94.53	87.99–101.54	17.00	98.83

Notes: AUC_0-t_, area under the plasma concentration–time curve from time 0 to the time of the last measurable concentration; AUC_0-∞,_ area under the plasma concentration–time curve from time 0 to infinity; C_max_, maximum plasma drug concentration; CV, coefficients of variation; GLSM, geometric least-squares mean; N, the bioequivalent analysis set population.

**FIGURE 3:**
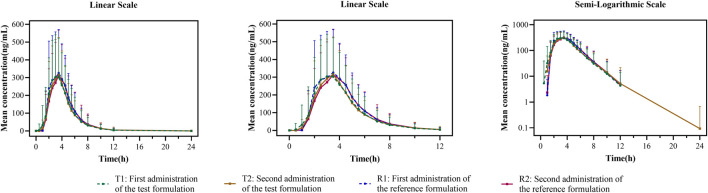
Mean plasma concentration–time curves for the test and reference formulations after a single dose of 20 mg of the esomeprazole enteric-coated tablet in healthy subjects under fed conditions. *N* = 40. Bars represent SD. T1: first administration of the test formulation; T2: second administration of the test formulation; R1: first administration of the reference formulation; R2: second administration of the reference formulation.

In the fed study, the data of 40 enrolled subjects were entered into the pharmacokinetic analysis. Figure 3 shows the mean plasma concentration–time curves of the two formulations under fed conditions. The changing trend of the four plasma concentration–time curves is also very similar. The primary pharmacokinetic analysis parameters are also listed in Table 2. For the test or reference formulation, C_max_ were 425.07 ± 220.29 and 467.04 ± 228.53 ng/mL, AUC_0-t_ were 1153.56 ± 746.72 and 1222.44 ± 758.19 ng h/m, AUC_0-∞_ were 1167.32 ± 760.77 and 1238.59 ± 775.71 ng h/m, and t_1/2_ were 1.04 ± 0.40 and 1.01 ± 0.36 h. The results of bioequivalence assessment of the fed study are shown in Table 4. The S_WR_ for C_max_, AUC_0-t_, and AUC_0-∞_ of reference formulation were 0.2473, 0.1708, and 0.1686, respectively, which were less than 0.294. As a result, the ABE method was selected to evaluate the bioequivalence. The 90% CIs of the GLSM ratios were 80.53%–94.95% for C_max_, 87.46%–97.26% for AUC_0-t_, and 87.46%–97.17% for AUC_0-∞_, which were also within the accepted bioequivalence range of 80.00%–125.00%. These results suggested that the two formulations of esomeprazole exhibited bioequivalence under fed conditions. The CV_w_% for C_max_, AUC_0-t_, and AUC_0-∞_ were 25.12%, 17.20%, and 16.98%, respectively, which further showed that esomeprazole does not show high variability, regardless of fasting or fed conditions.

**TABLE 4:** Bioequivalence assessment of pharmacokinetic parameters of esomeprazole after a single dose 20 mg of test and reference esomeprazole magnesium enteric-coated tablet in healthy Chinese subjects under fed conditions.

Parameters	Fed condition (*N* = 37)									
	Average bioequivalence (ABE)				Reference-scale average bioequivalence (RSABE)			Intra-subject CV (%)		Power (%)
	GLSM T	GLSM R	Ratio of GLSM (%)	90% CI (%)	S_WR_	Point estimate (0.8,1.25)	Criteria bound (≤0)	CV_wt_	CV_wr_	
C_max_	359.763	411.415	87.45	80.53–94.95	0.2473	0.8745	0.0013	31.39	25.12	61.85
AUC_0-t_	930.8549	1009.2660	92.23	87.46–97.26	0.1708	0.9223	−0.0045	23.47	17.20	99.45
AUC_0-∞_	940.8079	1020.5354	92.19	87.46–97.17	0.1686	0.9219	−0.0040	23.35	16.98	99.47

Notes: AUC_0-t_, area under the plasma concentration–time curve from time 0 to the time of the last measurable concentration; AUC_0-∞_, area under the plasma concentration–time curve from time 0 to infinity; C_max_, maximum plasma drug concentration; CV, coefficients of variation; GLSM, geometric least-squares mean; N, the bioequivalent analysis set population.

### Safety profile

In these two studies, the safety assessment set included a total of 72 subjects who received at least one dose of esomeprazole. In the fasting study, four AEs occurred in two subjects with an incidence of 6.3% (2/32). The four AEs were leukocyte count decrease, neutrophil count decrease, leukocyte count increase, and neutrophil count increase. The intensity of all the AEs was mild and graded as Grade 1. The outcome of all the AEs was recovered, and the subjects received no treatment. In the fed study, 17 AEs occurred in 11 subjects with an incidence of 27.5 (11/40). Among these AEs, eight AEs in five subjects were evaluated to be related to the study drug, including anemia, leukocyte count decrease, neutrophil count decrease, eosinophil count increase, fever, and blood bilirubin increase. In addition, three subjects did not complete the trial and withdrew early in the different treatment periods due to their respective AEs, including upper respiratory tract infection, fever, and chickenpox. No serious AEs occurred in both studies. These safety results showed that esomeprazole had good safety and was well-tolerated in healthy subjects under fasting and fed conditions.

## Discussion

Although esomeprazole has been gradually used to treat GERD and other gastric acid-related diseases since 2003, there is relatively little public information on the pharmacokinetics of esomeprazole enteric-coated tablets 20 mg, especially among Chinese people. The present study fully compared and evaluated the pharmacokinetics and bioequivalence between a generic formulation and the original drug of the esomeprazole enteric-coated tablet in healthy Chinese subjects under fasting and feeding conditions.

We compared the primary pharmacokinetic parameters of esomeprazole enteric-coated tablets 20 mg with those previously reported under fasting and fed conditions. Under the fasting condition, T_max_ and t_1/2_ of esomeprazole were about 2 and 1.2 h, respectively, similar to those in previous reports (Hassan-Alin et al., 2000; Hassan-Alin et al., 2005; AstraZeneca, 2022), which validated that the esomeprazole enteric-coated tablet is rapidly absorbed and eliminated after oral administration. However, in the present fasting study, the results of C_max_ and AUC of esomeprazole were different from those previously reported (Andersson et al., 2001; Sostek et al., 2007; Ullah et al., 2010), but were consistent with those found in the literature on Chinese subjects (Liu et al., 2020). The research subjects in the previous studies were of Caucasian ethnicity, while our research subjects were of Asian ethnicity. In our study, C_max_ was lower than that in the previous research results, but AUC was higher than that of the previous research results. The reason for the decrease in C_max_ is unclear, and it may be related to the lower body weight or BMI of the Chinese subjects in this study than that of the Caucasian subjects in previous studies. The possible reason for the differences in systemic drug exposure (as indicated by AUC) relates to esomeprazole’s main metabolic enzyme CYP2C19. As known, the frequency distribution of CYP2C19 gene polymorphism has obvious ethnic and regional differences. It is reported that the incidence of poor metabolizer (PM) of CYP2C19 in Caucasians is 3%–5%, while the incidence of PM in Asians is 13 %–23% (Gong et al., 2018; Tanaka T et al., 2019). The PM population has a higher plasma exposure due to the lower activity of CYP2C19 and slower drug clearance (Hokimoto et al., 2014). It has been reported in the literature that after repeated administration of esomeprazole 40 mg once a day, the average AUC of slow metabolizers is nearly 100% higher than that of individuals with active CYP2C19 (fast metabolizers) (AstraZeneca, 2022). Unfortunately, due to the lack of evaluation of the CYP2C19 genotype in this study, we cannot provide sufficient evidence to support this hypothesis, which is one of the limitations of this study.

Furthermore, we compared the pharmacokinetic profiles of esomeprazole under fasting and fed conditions in the two studies. Table 2 shows clearly that the absorption rate and absorption degree of esomeprazole were obviously affected by food, regardless of the use of test or reference products. When the subjects took esomeprazole enteric-coated tablets after high-fat breakfast, C_max_ and AUC of esomeprazole decreased by about 40%; meanwhile, T_max_ of esomeprazole was delayed from 2 to 3.5 h. Eating high-fat food may be the main factor causing these changes. As known, enteric-coated tablets are disintegrated and absorbed in the intestinal fluid. High-fat and high-calorie food can prominently reduce the rate of gastric emptying and prolong the time for the drug to reach the intestine tract (Deng et al., 2017), thus reducing the absorption rate and degree of the enteric-coated tablets. Therefore, compared with the fasting trial results, the T_max_ values of esomeprazole were longer under fed conditions, while the C_max_ and AUC values were lower. These results of the present study further supported the opinion that food can delay the absorption of esomeprazole and reduce its exposure in the human body (AstraZeneca, 2022).

## Conclusion

The present study demonstrated that the test formulation and reference formulation of esomeprazole enteric-coated tablets exhibited bioequivalence in healthy Chinese subjects. In addition, the two preparations were well-tolerated, and there were no major safety problems. The test formulation became the first generic drug of esomeprazole enteric-coated tablets of 20 mg in China. The two formulations can replace each other in clinical use, reducing the economic burden on patients with GERD and other acid-related diseases in China to a certain extent. In addition, the current research results can provide an important reference for developing generic esomeprazole enteric-coated tablets.

## Data Availability

The original contributions presented in the study are included in the article/Supplementary Material; further inquiries can be directed to the corresponding author.
