# Corrigendum: Pharmacokinetics and bioequivalence study of esomeprazole magnesium enteric-coated tablets 20 mg in healthy Chinese subjects under fasting and fed conditions

**DOI:** 10.3389/fphar.2023.1217008

**Published:** 2023-06-01

**Authors:** Ying Ding, Nannan Chu, Linling Que, Kai Huang, Yuanxing Chen, Wei Qin, Zhenzhong Qian, Yunfei Shi, Zhen Xu, Qing He

**Affiliations:** Wuxi People’s Hospital Affiliated to Nanjing Medical University, Wuxi, China

**Keywords:** esomeprazole magnesium enteric-coated tablet, bioequivalence, pharmacokinetics, safety, healthy Chinese subjects

In the published article, there was an error in the **author list**. The corrected author list appears below.

“Ying Ding†, Nannan Chu†, Linling Que, Kai Huang, Yuanxing Chen, Wei Qin, Zhenzhong Qian, Yunfei Shi, Zhen Xu, and Qing He^*”^

A correction has been made to **Author contributions**. This sentence previously stated:

“YD and QH designed the study. NC, YD, LQ, KH, YC, WQ, ZQ, YS, and ZX performed the clinical study. NC wrote and revised the manuscript.”

The corrected sentence appears below:

“QH and YD designed the study. YD, LQ, KH, YC, WQ, ZQ, YS, and ZX performed the clinical study. NC wrote and revised the manuscript.”

The authors apologize for this error and state that this does not change the scientific conclusions of the article in any way. The original article has been updated.

